# Behavioral economics, lifestyle, and health-related factors associated with participation in breast and cervical cancer screenings: A cross-sectional analysis of Japanese women

**DOI:** 10.1016/j.pmedr.2025.103249

**Published:** 2025-09-15

**Authors:** Miho Satoh, Naoko Sato, Mizuki Sekino

**Affiliations:** a Department of Fundamental Nursing, Yokohama City University, Graduate School of Medicine, 3-9 Fukuura, Kanazawa-Ku, Yokohama, Kanagawa 236-0004, Japan; b Department of Adult and Gerontological Nursing, Fukushima Medical University, 1 Hikarigaoka, Fukushima City, Fukushima 960-1295, Japan

**Keywords:** Cancer screening, Cervical cancer, Breast cancer, Lifestyle, Behavioral economics

## Abstract

**Objective:**

This study investigated factors influencing Japanese women's participation in breast and cervical cancer screenings, with a focus on health behaviors, behavioral economics characteristics, socioeconomic status, and physical and mental health.

**Methods:**

Using secondary data from the Japan Household Panel Survey (Wave 2021, collected February 2021), we analyzed responses from 410 women aged ≤70 years. Key variables included risk aversion, time preference, exercise frequency, smoking status, alcohol consumption, nutritional intake, subjective and mental health, body mass index, and sociodemographic factors such as education, employment, and municipality type.

**Results:**

The participants' mean age was 52.92 years (standard deviation = 9.17 years). Of the women, 15.9 % underwent cervical cancer screening and 16.8 % underwent breast cancer screening. A logistic regression analysis revealed that, for both breast and cervical cancer screenings, high psychological distress, risk aversion, and smoking were associated with lower participation, whereas regular exercise, permanent employment, and residence in towns and villages were associated with higher participation. Smoking was significantly associated with breast cancer screening participation, and utilization of medical services was significantly associated with cervical cancer screening participation.

**Conclusion:**

Interventions incorporating behavioral economics approaches, such as addressing risk perception and promoting health behaviors, may enhance Japanese women's cancer screening participation.

## 1 Introduction

Japan's recently rising mortality rates for cervical and breast cancer might be attributable to persistently insufficient screening rates for these cancers. The screening rate is 47.4 % for breast cancer and 43.6 % for cervical cancer among Japanese women—considerably lower than the rates of 70 % for both cancers in other developed countries, such as the US, the UK, and Finland (OECD, 2024). These international comparisons suggest that Japan may be missing opportunities to avoid preventable deaths from cancer through screenings, and addressing this issue is an urgent task.

Previous studies in Japan and internationally have shown that cancer screening participation is influenced by health awareness, health behaviors, lifestyle, education, and socioeconomic status (Castañeda et al., 2023; Wakui et al., 2014).

Lower socioeconomic status, financial and time constraints, and limited health literacy are also associated with lower screening rates (Shimoda et al., 2021; Vallone et al., 2022). While recommendations from healthcare providers, sending of screening invitations, and government coverage of screening costs encourage participation in screenings (Vallone et al., 2022), psychological barriers may impede participation. These barriers include fear of cancer, anxiety about the screening or treatment methods, discomfort or embarrassment with the process, limited knowledge or understanding of cancer and screenings, and family responsibilities such as housework, childcare, and caregiving (Mon et al., 2024).

Additionally, behavioral economics has highlighted that people often make irrational health decisions, avoiding screenings due to anxiety or biases such as present bias or loss aversion (Emanuel et al., 2015; Zhao and Liu, 2021). However, quantitative research on these factors in cancer screening remains limited. Understanding such decision-making tendencies may support more effective screening strategies.

This study sought to analyze factors related to participation in breast and cervical cancer screenings among Japanese women from multiple perspectives: health behaviors, the characteristics of behavioral economics, socioeconomic aspects, and physical and mental health.

## 2 Methods

### 2.1 Data source and participants

This study was a secondary analysis of nationally representative data from the Japanese Household Panel Survey (JHPS/KHPS), administered by the Panel Data Research Center at Keio University (Keio University, n.d). The surveys employ stratified two-stage probability sampling to construct survey panels and have been conducted annually since 2004, with an additional panel introduced in 2009.

Data from Wave 2021, supplemented with demographic information from Waves 2004 and 2009, were analyzed. The analytical sample comprised 410 women aged ≤70 years in 2021 who were eligible for breast and cervical cancer screening in Japan and had complete data available for all variables of interest.

Table 1 summarizes the participants' characteristics. The mean age was approximately 53 years; 77 % were married, and 70 % had a child aged ≥15 years. Caregiving responsibilities were reported by approximately 23 %. Educational attainment was distributed across junior/senior high school (43–46 %), junior/technical college (29–35 %), and university/graduate school (19–26 %). Employment was permanent for 22–34 %, temporary for 46–49 %, and self-employed, homemaker, or unemployed for 26–30 %. Most participants resided in urban areas, and 64–77 % had visited a medical facility or been hospitalized in the past year.

**Table 1:** Health Behaviors, Behavioral Economic Characteristics, Physical and Mental Health, and Sociodemographic of Participants According to Cervical and Breast Cancer Screening Status Among Japanese Women Aged ≤70 Years (*n* = 410) from the Japanese Household Panel Survey, Wave 2021.

		Cervical Cancer Screening			Breast Cancer Screening		
		No (*n* = 345)	Yes (*n* = 65)	*p*-value^†^	No (*n* = 341)	Yes (*n* = 69)	p-value^††^
		n (%)/mean (SD)	n (%)/mean (SD)		n (%)/mean (SD)	n (%)/mean (SD)	
Marital Status	Married	266 (77.1)	50 (76.9)	0.98	262 (76.8)	54 (78.3)	0.80
	Unmarried	79 (22.9)	15 (23.1)		79 (23.2)	15 (21.7)	
Child Status	None	48 (13.9)	11 (16.9)	0.59	49 (14.4)	10 (14.5)	0.46
	<15-year-old	47 (13.6)	11 (16.9)		45 (13.2)	13 (18.8)	
	≥15-year-old	250 (72.5)	43 (66.2)		247 (72.4)	46 (66.7)	
Caregiver Role Status	No	270 (78.3)	49 (75.4)	0.61	268 (78.6)	51 (73.9)	0.39
	Yes	75 (21.7)	16 (24.6)		73 (21.4)	18 (26.1)	
Educational Attainment	Junior/ Senior high school	160 (46.4)	28 (43.1)	0.86	157 (46.0)	31 (44.9)	0.27
	Junior/ Technical college	119 (34.5)	23 (35.4)		122 (35.8)	20 (29.0)	
	University/Graduate School	66 (19.1)	14 (21.5)		62 (18.2)	18 (26.1)	
Employment Status	Permanent	75 (21.7)	22 (33.8)	0.07	76 (22.3)	21 (30.4)	0.35
	Temporary	166 (48.1)	30 (46.2)		166 (48.7)	30 (43.5)	
	Self-employed/Homemaker/ Unemployed	104 (30.1)	13 (20.0)		99 (29.0)	18 (26.1)	
Alcohol Consumption	<180 mL / week	180 (52.2)	38 (58.5)	0.35	174 (51.0)	44 (63.8)	0.05
	≥180 mL / week	165 (47.8)	27 (41.5)		167 (49.0)	25 (36.2)	
Smoking Status	Never smoker	229 (66.4)	43 (66.2)	0.85	227 (66.6)	45 (65.2)	0.45
	Former smoker	81 (23.5)	14 (21.5)		81 (23.8)	14 (20.3)	
	Current smoker	35 (10.1)	8 (12.3)		33 (9.7)	10 (14.5)	
Utilization of medical services in the Past Year	No	121 (35.1)	15 (23.1)	0.06	123 (36.1)	13 (18.8)	0.01
	Yes	224 (64.9)	50 (76.9)		218 63.9	56 (81.2)	
Municipality Type	Government-designated city⁎	111 (32.2)	17 (26.2)	0.16	106 (31.1)	22 (31.9)	0.24
	City⁎⁎	202 (58.6)	37 (56.9)		203 (59.5)	36 (52.2)	
	Towns or villages⁎⁎⁎	32 (9.3)	11 (16.9)		32 (9.4)	11 (15.9)	
Age		53.03 (9.24)	52.32 (8.85)	0.57	52.50 (9.14)	54.97 (9.10)	0.04
Annual Household Income (million JPY [thousand EUR])^1^		737.67 (480.91) [58.10 (37.90)]	827.22 (476.49) [65.10 (37.50)]	0.17	743.29 (470.75) [58.50 (37.10)]	794.23 (529.03) [62.60 (41.70)]	0.42
Subjective Health^2^		3.45 (0.88)	3.43 (0.85)	0.88	3.46 (0.89)	3.39 (0.79)	0.57
Mental Health^3^		5.03 (4.49)	4.34 (4.15)	0.25	5.05 (4.59)	4.25 (3.58)	0.17
Sleep Duration^4^		6.75 (0.99)	6.67 (0.86)	0.52	6.76 (0.99)	6.66 (0.85)	0.43
Risk Aversion^5^		47.58 (16.30)	43.38 (16.33)	0.06	47.76 (16.31)	42.75 (16.08)	0.02
Time Preference Rate^6^		0.16 (0.13)	0.16 (0.14)	1.00	0.16 (0.13)	0.18 (0.14)	0.38
Weekly Frequency of Exercise^7^		0.70 (1.46)	1.22 (1.88)	0.01	0.69 (1.44)	1.20 (1.91)	0.04
Body Mass Index^8^		22.36 (3.88)	22.26 (3.66)	0.85	22.36 (3.85)	22.26 (3.80)	0.85
Protein Intake^9^		2.49 (0.99)	2.43 (0.92)	0.64	2.48 (0.95)	2.48 (0.98)	0.97
Vegetable Intake^9^		2.67 (1.14)	2.46 (1.03)	0.17	2.65 (1.14)	2.57 (1.09)	0.55
Fruits Intake^9^		4.75 (1.74)	4.58 (1.56)	0.48	4.78 (1.72)	4.45 (1.69)	0.14

† Calculated using the chi-square test for categorical variables.

†† Calculated using the unpaired t-tests for continuous variables.

1. Self-reported, continuous, annual household income in JPY was converted to approximate EUR using the average exchange rates for 2021 (1 EUR ≈ 127 JPY). 2. Single 5-point Likert scale. 3. Kessler Psychological Distress Scale, six items rated on a 5-point scale; total score 0–24. 4. Self-reported average hours per night. 5. Assessed by a hypothetical rainfall question; higher scores = greater aversion. 6. Measured by delayed monetary choice; 8-point scale (−5 % to 40 %). 7. Self-reported weekly frequency. 8. Calculated from self-reported height and weight. 9. frequency of consumption recorded as “three times a day,” “twice a day,” “once a day,” “4–6 times a week,” “2–3 times a week,” “once a week,” “less than once a week to 3 times a month,” or “never,” and scored on an 8-point scale from 1 (least frequent) to 8 (most frequent).

EUR, euro; JPY, Japanese yen; SD, standard deviation.

⁎ Population ≥ 500,000.

⁎⁎ Population ≥ 50,000 to <500,000.

⁎⁎⁎ Population < 50,000.

### 2.2 Variables

The dependent variables in this study were participation in breast cancer screening and participation in cervical cancer screening, with each treated as a binary variable (screened vs. not screened) and measured as whether the respondent underwent screening in 2020.

Independent variables comprised health behaviors, behavioral economics, health status, and sociodemographic characteristics. Health behaviors included exercise frequency, sleep duration, alcohol consumption, smoking status, nutritional intake, and healthcare utilization. Alcohol consumption was classified as <180 mL/week or ≥ 180 mL/week, based on national guidelines (Ministry of Health, Labour and Welfare, 2024). Smoking was categorized as never, former, or current. Nutritional intake was measured as the frequency of protein, vegetable, and fruit consumption over the past month, recorded on a continuous scale: “three times a day,” “twice a day,” “once a day,” “4–6 times a week,” “2–3 times a week,” “once a week,” “less than once a week to 3 times a month,” and “never” were treated as continuous variables and scored on an eight-point scale from 1 (least frequent) to 8 (most frequent). These frequencies reflect not only food intake but also broader dietary patterns, health behaviors, and lifestyle habits (Wakai, 2009). Utilization of medical services in the past year was categorized as “yes” or “no.”

Behavioral economic factors included risk aversion and time preference, treated as continuous variables. Risk aversion was assessed by a question on expected rainfall, with higher scores indicating greater aversion (Tsutsui et al., 2012). Time preference was measured by asking, “Instead of receiving 10,000 yen in one month, how much would you need in 13 months to wait?” rated on an eight-point scale (−5 % to 40 %) (Picone et al., 2004).

Health status was measured using several indicators as continuous variables: subjective health was assessed on a five-point Likert scale from “good” to “poor”; mental health was evaluated using the Kessler Psychological Distress Scale (Kessler et al., 2002), which comprises six items measuring depressive and anxiety symptoms in the past month on a five-point scale, yielding a total score between 0 and 24. Body mass index was calculated from self-reported height and weight.

Sociodemographic variables included age, marital status (single or married), parental status (none, having a child aged <15 years, or having a child aged ≥15 years), caregiving role status (yes or no), education (junior/senior high school, junior/technical college, or university/graduate school), employment (permanent, temporary, self-employed/homemaker/unemployed), municipality type (government-designated city, city, or town/village), and household income.

### 2.3 Statistical analyses

Descriptive statistics were calculated for all variables. Subsequently, chi-squared tests, adjusted standardized residuals, and unpaired *t*-tests were employed to examine the associations of breast cancer and cervical cancer screening participation with daily health behaviors, risk aversion, time preference rate, health status, and sociodemographic variables. To identify factors that influence participation in breast and cervical cancer screenings, logistic regression analyses were performed.

We conducted all statistical analyses using SPSS Statistics 29.0 for Mac. Statistical significance was set at *p* < 0.05 (two-tailed).

### 2.4 Ethical considerations

As the JHPS/KHPS data are deidentified data, an ethics review was deemed unnecessary. JHPS/KHPS participants consented to data use, anonymity, confidentiality, and secondary use by the Panel Data Research Center at Keio University.

## 3 Results

Participants' demographic characteristics are shown by screening status in Table 1. Participants who underwent cervical cancer screening reported significantly higher weekly exercise frequency compared to those not screened (1.22 vs. 0.70 times/week, *p* = 0.01). Participants who underwent breast cancer screening were older (54.97 vs. 52.50 years, *p* = 0.04), exercised more frequently (1.20 vs. 0.69, p = 0.04), had lower risk aversion (42.75 vs. 47.76, *p* = 0.02), and were more likely to have utilized medical services in the past year (81.2 % vs. 63.9 %, p = 0.01).

Table 2 presents factors associated with participation in cervical and breast cancer screenings. For cervical cancer screening, higher psychological distress (OR = 0.93, 95 % CI = 0.86, 0.99) and greater risk aversion (OR = 0.98, 95 % CI = 0.96, 0.99) were significantly associated with lower odds of screening participation. By contrast, higher exercise frequency (OR = 1.25, 95 % CI = 1.05, 1.48) and permanent employment (OR = 2.95, 95 % CI = 1.22, 7.16) were positively associated with screening participation. Furthermore, residing in a government-designated city (OR = 0.26, 95 % CI = 0.10, 0.69) or a city (OR = 0.35, 95 % CI = 0.15, 0.84) was associated with significantly lower participation compared with residing in towns or villages.

**Table 2:** Multivariable Logistic Regression Analysis of Behavioral Economic Characteristics, Health-Related Behaviors, Physical and Mental Health, and Sociodemographic Associated with Participation in Cervical and Breast Cancer Screening Among Japanese Women Aged ≤70 years (n = 410) from the Japanese Household Panel Survey, Wave 2021.

		Cervical cancer screening		Breast cancer screening	
		Exp(B)	95 % CI	Exp(B)	95 % CI
Age		0.98	0.94, 1.01	1.01	0.98, 1.05

Marital Status (ref = Unmarried)					
	Married	1.17	0.51, 2.70	1.46	0.64, 3.31

Child Status (ref = None)					
	<15 years old	0.81	0.34, 1.96	1.45	0.50, 4.22
	≥15 years old	1.21	0.41, 3.56	1.03	0.43, 2.49

Caregiver Role Status (ref = No)					
	Yes	1.30	0.65, 2.59	1.21	0.62, 2.35

Educational Attainment (ref = University/Graduate school)					
	Junior/ Senior high school	0.89	0.38, 2.09	0.60	0.27, 1.34
	Junior/ Technical college	1.04	0.46, 2.37	0.56	0.25, 1.25

Employment Status (ref = Homemaker/Unemployed/Self-employed)					
	Permanent	2.95	1.22, 7.16	2.65	1.12, 6.31
	Temporary	1.73	0.78, 3.85	1.44	0.68, 3.05

Alcohol Consumption (ref ≤180 mL/week)					
	≥180 mL/week	0.76	0.42, 1.40	0.64	0.35, 1.18

Smoking Status (ref = Current smoker)					
	Never smoker	0.44	0.16, 1.21	0.28	0.10, 0.74
	Former smoker	0.49	0.17, 1.43	0.33	0.12, 0.92

Utilization of Medical Services in the Past Year (ref = No)					
	Yes	1.86	0.92, 3.76	2.18	1.06, 4.47
Protein Intake^1^		1.24	0.84, 1.84	1.21	0.83, 1.78
Vegetable Intake^1^		0.81	0.56, 1.16	0.93	0.66, 1.31
Fruits Intake^1^		1.00	0.83, 1.20	0.95	0.79, 1.14
Subjective Health^2^		0.79	0.55, 1.12	0.77	0.54, 1.09
Mental Health^3^		0.93	0.86, 0.99	0.92	0.85, 0.99
Sleep Duration^4^		0.94	0.68, 1.28	0.94	0.69, 1.29
Risk Aversion^5^		0.98	0.96, 0.99	0.98	0.96, 0.99
Time Preference Rate^6^		1.43	0.17, 11.91	3.13	0.40, 24.87
Weekly Frequency of Exercise^7^		1.25	1.05, 1.48	1.19	1.01, 1.42
Body Mass Index^8^		0.99	0.91, 1.07	0.99	0.91, 1.07
Annual Household Income (million JPY [thousand EUR])^9^		1.00	1.00, 1.00	1.00	1.00, 1.00

Municipality Type (ref = Towns or villages⁎⁎⁎)					
	Government-designated city⁎	0.26	0.10, 0.69	0.32	0.12, 0.85
	City⁎⁎	0.35	0.15, 0.84	0.30	0.13, 0.74

1. Frequency of consumption recorded as “three times a day,” “twice a day,” “once a day,” “4–6 times a week,” “2–3 times a week,” “once a week,” “less than once a week to 3 times a month,” or “never,” and scored on an 8-point scale from 1 (least frequent) 8 (most frequent). 2. Single 5-point Likert scale. 3. Kessler Psychological Distress Scale, six items rated on a 5-point scale; total score 0–24. 4. Self-reported average hours per night. 5. Assessed by a hypothetical rainfall question; higher scores = greater aversion. 6. Measured by delayed monetary choice; 8-point scale (−5 % to 40 %). 7. Self-reported weekly frequency. 8. Calculated from self-reported height and weight. 9 Self-reported, continuous, annual household income in JPY was converted to approximate amounts in EUR using the average exchange rates for 2021 (1 EUR ≈ 127 JPY).

CI, confidence interval; EUR, euro; JPY, Japanese yen.

⁎ Population ≥ 500,000.

⁎⁎ Population ≥ 50,000 to <500,000.

⁎⁎⁎ Population < 50,000.

For breast cancer screening, higher psychological distress (OR = 0.92, 95 % CI = 0.85, 0.99) and greater risk aversion (OR = 0.98, 95 % CI = 0.96, 0.99) were inversely associated with screening participation. Conversely, higher exercise frequency (OR = 1.19, 95 % CI = 1.01, 1.42) and permanent employment (OR = 2.65, 95 % CI = 1.12, 6.31) were significantly associated with increased screening participation. Notably, women who had never smoked (OR = 0.28, 95 % CI = 0.10, 0.74) or formerly smoked (OR = 0.33, 95 % CI = 0.12, 0.92) were less likely to participate in screening compared with current smokers. Additionally, women who had utilized medical services in the past year were significantly more likely to undergo breast cancer screening (OR = 2.18, 95 % CI = 1.06, 4.47). Finally, residing in a government-designated city (OR = 0.32, 95 % CI = 0.12, 0.85) or a city (OR = 0.30, 95 % CI = 0.13, 0.74) was associated with lower screening participation than residing in towns or villages.

## 4 Discussion

This study revealed that higher levels of psychological distress, greater risk aversion, and smoking status were associated with reduced screening participation, whereas regular exercise, permanent employment, and residence in towns or villages were associated with increased screening participation.

The finding that higher psychological distress was associated with lower participation in breast and cervical cancer screenings was consistent with prior research indicating that poor mental health may hinder engagement in preventive health behaviors (Kerrison et al., 2023). Psychologically vulnerable individuals may delay screening due to heightened anxiety and fear. Moreover, greater risk aversion was linked to a lower screening rate, suggesting that, despite its typical association with preventive behavior, risk aversion may lead to information avoidance when negative outcomes are anticipated. This aligns with evidence that individuals avoid threatening information (Zhao and Liu, 2021) and are influenced by cognitive biases such as loss aversion and status quo bias, which may deter cancer screening (Goldzahl, 2017).

Breast and cervical cancer screening rates were significantly higher among women who regularly exercised. This indicates that health behaviors tend to co-occur synergistically and that autonomous motivation for health may be higher (Ng et al., 2012). Women who had never or formerly smoked were less likely to be screened for breast cancer than women who were current smokers, in contrast to previous findings (Eng et al., 2020). This may reflect non-smokers' health optimism and low awareness of the need for screening (Sheeran et al., 2014), while smokers may be getting screened due to increased risk awareness.

For both breast and cervical cancer screenings, permanently employed women had significantly higher rates of screening participation than those who were unemployed/homemakers/self-employed. This suggests that employment status may affect access to health resources and information. The presence or absence of workplace-based screenings and opportunities for health education in the workplace may influence health behavior. This could also be interpreted as a form of inequality related to screening behavior, stemming from disparities in pathways such as health insurance coverage, economic stability, and the availability of workplace health promotion support (Mon et al., 2024)—an issue deserving further attention.

Women who had utilized medical services in the past year showed significantly higher participation in breast cancer screening, suggesting that healthcare encounters may be a key opportunity for screening recommendations and information.

There were also residential-area differences, with residents of government-designated and mid-sized cities having lower screening rates than those of towns and villages. Although urban residents may have better access to medical facilities, attributes such as a fast-paced lifestyle, greater anonymity, and information overload may weaken their motivation to seek out screenings. In contrast, people in rural areas may benefit from stronger community ties and more effective outreach efforts from local governments (Huang and Ekenga, 2025).

These results highlight the complex interplay of psychological, behavioral, and social factors that influence cancer screening rates. Interventions incorporating behavioral economics, stigma-free support, community-based approaches, and flexible screening options are essential for promoting cancer screening participation. To reach diverse populations with diverse values, integrated, multilayered strategies that address these factors are essential.

This study has some limitations. Its cross-sectional design precludes causal inference, warranting longitudinal and interventional research. Self-administered questionnaires may have introduced bias; future studies should clarify individuals' perceptions and motivations regarding screenings. Screening behavior was treated as binary, without considering screening type, continuity, or frequency; linking registry and medical record data could enhance validity. Importantly, the modest sample size may have limited statistical power and precision, particularly for rare categories, and the inclusion of numerous variables raises the risk of overfitting.

## 5 Conclusion

For breast cancer screening and cervical cancer screening, high psychological distress, risk aversion, and never or formerly smoking were associated with lower participation, whereas regular exercise, permanent employment, and rural residence were associated with higher participation. Additionally, smoking status was significantly associated with breast cancer screening participation, and utilization of medical services was significantly associated with cervical cancer screening participation.

## CRediT authorship contribution statement

**Miho Satoh:** Writing – original draft, Validation, Software, Resources, Project administration, Methodology, Investigation, Formal analysis, Data curation, Conceptualization. **Naoko Sato:** Writing – review & editing, Methodology, Investigation, Conceptualization. **Mizuki Sekino:** Writing – review & editing, Conceptualization.

## Funding

This research did not receive any specific grant from funding agencies in the public, commercial, or not-for-profit sectors.

## Declaration of competing interest

The authors declare that they have no known competing financial interests or personal relationships that could have appeared to influence the work reported in this paper.

## Data Availability

The third-party dataset (Approval No. 5841: Data ID 229 JHPS/KHPS2004–2022) supporting the conclusions of this article is available from the Panel Data Research Center at Keio University with the approval of this organization (https://www.pdrc.keio.ac.jp/en) for researchers who meet the criteria for access to confidential data. The authors confirm that they did not have any special access privileges that others would not have. The authors also confirm that others would be able to access these data in the same manner as themselves.
